# Analysis of Hot Tensile Fracture and Flow Behaviors of Inconel 625 Superalloy

**DOI:** 10.3390/ma17020473

**Published:** 2024-01-19

**Authors:** Xin-Zhe Pan, Xiao-Min Chen, Meng-Tao Ning

**Affiliations:** 1 International Institute of Engineering, Changsha University of Science and Technology, Changsha 410114, China; panxinzhecsust@163.com; 2 College of Automotive and Mechanical Engineering, Changsha University of Science and Technology, Changsha 410114, China; ningmengtao6615@163.com

**Keywords:** superalloy, tensile, fracture morphology, constitutive model

## Abstract

In this work, Inconel 625 alloy is explored regarding high-temperature tensile deformation and fracture behaviors at a strain rate of 0.005–0.01 s^−1^ under a deformation temperature ranging from 700–800 °C. The subsequent analysis focuses on the impact of deformation parameters on flow and fracture characteristics. The fractured surface reveals that ductile fracture is dominated by the nucleation, growth, and coalescence of microvoids as the primary failure mechanisms. The elevated deformation temperature and reduced strain rate stimulate the level of dynamically recrystallized (DRX) structures, resulting in intergranular fractures. The Arrhenius model and the particle swarm optimization-artificial neural network (PSO-ANN) model are developed to predict the hot tensile behavior of the superalloy. It indicates that the PSO-ANN model exhibits a correlation coefficient (*R*) as high as 0.9967, surpassing the corresponding coefficient of 0.9344 for the Arrhenius model. Furthermore, the relative absolute error of 9.13% (Arrhenius) and 1.85% (PSO-ANN model) are recorded. The developed PSO-ANN model accurately characterizes the flow features of the Inconel 625 superalloy with high precision and reliability.

## 1. Introduction

Nickel-based superalloys are preferred in aircraft engines and various critical components of industrial gas turbines. Hot forming techniques e.g., extrusion, rolling, and forging are utilized for producing nickel-based superalloy components, which exhibit outstanding corrosion resistance, excellent processing performance, weldability, and high service temperature. However, successive plastic deformations like tension, torsion, and compression can occur in the materials during hot forming. In particular, hot tensile deformation conditions can generate fractures, voids, cracks, or fractures, consequently affecting the thermal-mechanical behavior of these components [1,2]. The high-temperature flow behavior of nickel-based alloys can disclose the underlying damage mechanisms. Thus, it is crucial to examine the hot tensile behavior of nickel-based superalloys.

The constitutive model can quantitatively realize the true stress-strain data during the deformation of materials, i.e., a productive approach to reproduce the high-temperature tensile behavior of metallic. For instance, Chen’s group [3] explored the hot tensile characteristics of GH4169 superalloy by using Arrhenius and PSO-ANN models. Considering the mean absolute relative error, correlation coefficient, root-mean-square error, and distribution of relative errors, they demonstrated the considerable predictive accuracy of the PSO-ANN model. Liu et al. [4] established the modified Zerilli−Armstrong, Johnson−Cook, and Arrhenius models for the tensile flow characteristics of the C-276 alloy and disclosed the superior accuracy of the modified Arrhenius model. Later on, the impact of deformation parameters on the thermal deformation behavior of a Ni-based superalloy was investigated through a stacked auto-encoders network model, which effectively characterized the high-temperature rheological behavior [5]. Wen et al. [6] explored a constitutive model based on unified dislocation density and reported the evolution of alloy phase transition and damage mechanisms through the thermal deformation characteristics of the Ti_6_Al_4_V alloy at a wide temperature and strain range. An appropriate constitutive model can establish the fundamental basis for precisely characterizing the tensile behavior of metals and alloys, which can provide accurate finite element simulations. Among the flow constitutive models, the phenomenological constitutive model is opted for widely due to simple form and strong intuitiveness in the counterpart of the physics-based and artificial neural network-based types [7]. The Johnson-Cook model endows its primary application: the rheological behavior under high strain, high strain rate, and elevated temperatures, but constrained applicability within certain limits. The Arrhenius model has three forms: exponential, power-law, and hyperbolic sinusoidal, and it can describe the plastic deformation behavior of materials in the steady-state stage. However, due to excessive parameters and lack of the strain-softening phenomenon in the deformation process, its steady state requires the stress value to be determined first [8]. Additionally, complex mechanisms occur during hot deformation, including DRX, dynamic recovery (DRV), work hardening (WH), adiabatic shear, and phase transformation [9]. Artificial intelligence models are favorable due to their strong nonlinear mapping capabilities, wide application range, and high prediction accuracy. Qiao et al. [10] established a support vector machine model and predicted the rheological behaviors of the AlCrFeNi multi-component alloy. Zhu et al. [11] developed a predictive constitutive model for the Inconel 925 superalloy by employing the ANN model in conjunction with genetic algorithm (GA) algorithms. A few studies explored the microstructure and flow behavior of the Inconel 625 superalloy during high-temperature deformation. Gao et al. [12] explored the mechanical properties of the Inconel 625 alloy and reported the different performances of DRX and static recrystallization at the grain and sub-grain boundaries. The deformation temperature has a significant influence on the nucleation mechanisms of DRX at different deformation stages [13]. The intermetallic precipitates dominate the mechanical properties of the material, and grain coarsening can boost hardness [14]. Guillen et al. [15] compared the tensile properties of the Inconel 625 alloy by hot isostatic pressing and forging, and the grain level stress of the deformed alloy is more uneven in the loading direction. Kim et al. [16] compared the difference between the precipitates in the laser powder bed melting 625 alloy and the forged 625 alloy and found that HIPed LPBF and deformed alloys show similar microstructure characteristics in terms of the average grain size, defects, special grain boundary ratio, and grain morphology. However, the higher sulfur content in the HIPed 625 superalloy results in lower fatigue durability at 650 °C.

The fracture mechanism of materials is intricately connected to the evolution of their rheological behavior and microstructure [17]. Kong et al. [18] employed hot tensile tests using the Inconel 625 alloy and disclosed the plastic rheological behavior via work hardening to hot softening. The coarsening of δ phases generated microvoids at the phases/matrix interfaces with rising tensile strain, which can reduce the reinforcing impact of δ phases [19]. Further, the stress concentration induced by Nb-rich phases led to crack initiation at temperatures below 650 °C [20]. The primary fracture mechanism of the Ni-based alloy involves microporous coalescence and intergranular fracture induced by DRX [3]. Liu et al. [21] examined a nickel-based superalloy comprised of a polycrystalline structure through a tensile test. An expedited degradation of the microstructure with rising temperature caused a notable decline in its mechanical properties. Zhang et al. [22] elucidated that ductility deterioration in the Inconel 718 superalloy was attributed to a combination of necking interactions, oxidation, and microstructural instability induced by cold-rolling.

Despite several studies on the hot tensile feature of Nickel-based superalloy, an accurate model has been lacking to reproduce the hot tensile characteristics of the Ni-based alloy precisely, as well as high effectiveness and ease of applications. To overcome the mentioned problem, a comprehensive study of nickel-based alloys is carried out. A series of hot tensile tests were performed using the Inconel 625 alloy. The objective includes a focus on exploring tensile characteristics and fracture mechanisms through scanning electron microscopy (SEM). Two distinct models were developed to simulate the high-temperature tensile flow behavior of the Inconel 625 alloy. The models were verified through experimental results to assess their effectiveness and predictive capability.

## 2. Experimental Methodologies

**Figure 1:**
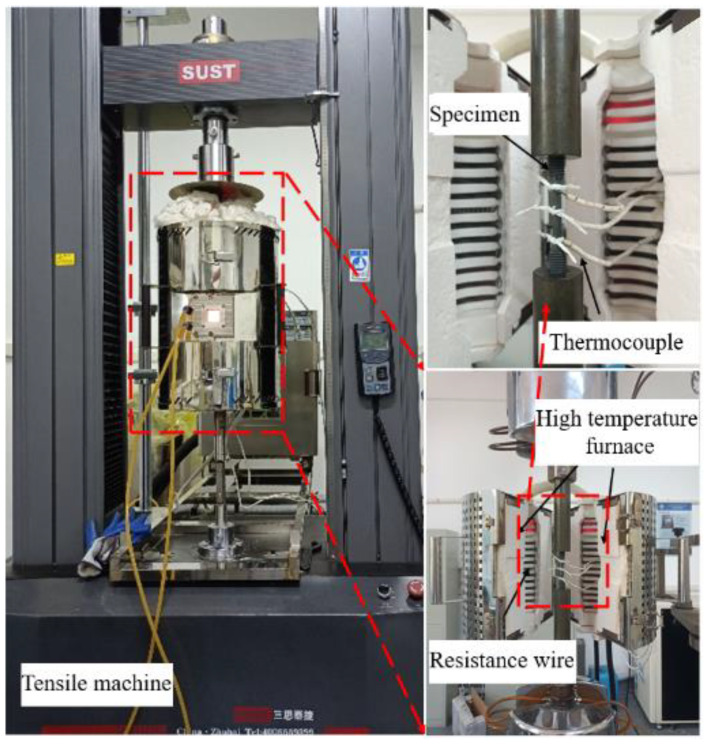
Tensile machine.

The commercially accessible Inconel 625 superalloy was utilized. The nominal composition of the alloy is listed in Table 1. The raw material underwent vacuum arc remelting (VAR), followed by hot forging to eliminate the casting structure. According to ISO 6892-2 [23], the material was processed into a cylindrical specimen with a diameter of 5 mm and a gauge length of 30 mm. Tensile tests were performed utilizing a CMT-5105GL tensile machine, manufactured by SUST company, Zhuhai city, China, as shown in Figure 1. Tests were carried out at deformation temperatures 700–800 °C with a rising step of 50 °C and strain rates of 0.0005, 0.001, 0.005, and 0.01 s^−1^. Preceding the initiation of the test, each sample was heated to the designated test temperature at a controlled rate of 10 °C/min, then retained for 15 min to ensure uniform thermal distribution within the sample. Ultimately, the tested sample underwent stretching until the fracture occurred. An average of three tensile tests was considered to elevate the precision of measurements.

**Figure 2:**
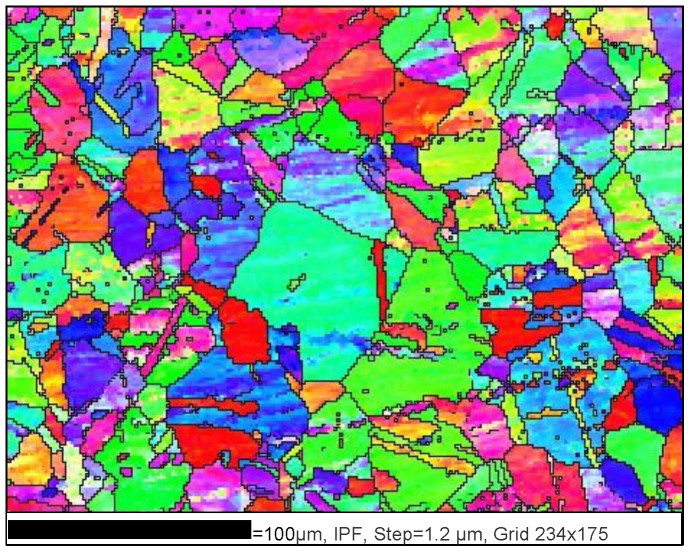
As-received microstructure of the tested superalloy.

SEM was used to examine the morphology of the fracture surfaces of the failed specimens. These samples were cut perpendicular to the stretched axis, originating from the center of each specimen. The microstructure of the longitudinal section near the fracture under different deformation parameters was observed using EBSD. The detailed procedure to prepare samples for SEM and EBSD can be seen in Refs. [3,24]. Figure 2 depicts the microstructure of the examined superalloy, which is consistent as reported in Ref. [25]. The original microstructure shows equiaxed grains and annealing twins exhibiting straight and lamella-like features. The formation of annealing twins is induced by the movement of grain boundaries resulting from internal stress during the hot forming and its following heat treatment. This causes the grains to rearrange and the grain boundaries to overlap [26]. The mean grain size is approximately 45 µm.

## 3. Results and Discussion

### 3.1. Hot Tensile Flow Curves

**Figure 3:**
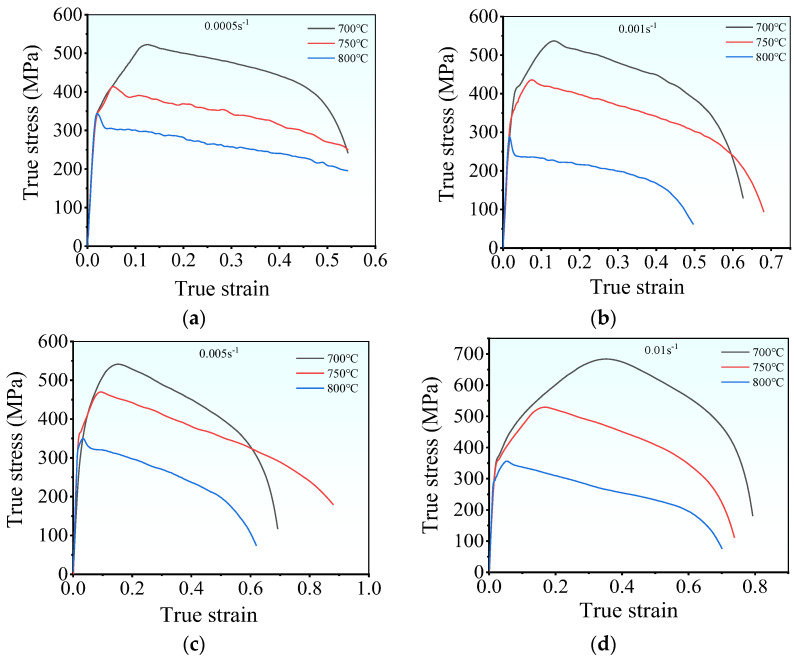
Hot tensile flow curves under the strain rates of: (**a**) 0.0005 s^−1^; (**b**) 0.001 s^−1^; (**c**) 0.005 s^−1^; (**d**) 0.01 s^−1^.

**Figure 4:**
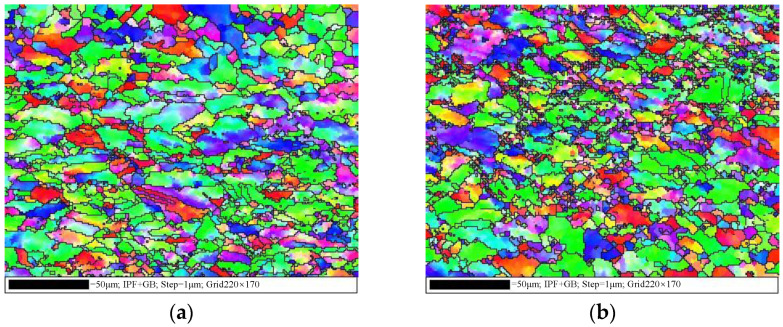
IPF maps of the longitudinal section near the fracture at (**a**) 700 °C, 0.001 s^−1^; (**b**) 800 °C, 0.01 s^−1^.

Figure 3 illustrates the hot tensile flow curves of the examined superalloy under various deformation conditions. The flow curves exhibit three distinct stages: elastic, plastic, and local necking stages [27]. During the initial elastic deformation stage, the rapid multiplication and entanglement of dislocations induce WH, leading to a prompt increase in flow stress that follows Hooke’s law [28,29]. In this stage, the occurrence of DRX is challenging, and relying only on DRV makes it difficult to compete with WH [18]. As strain increases, the material enters the plastic stage. Dislocation density gradually rises, which triggers DRX and consequently reduces the multiplication rate of dislocations [30,31]. The rate of the tensile stress increases slowly with intensifying strain. This is because DRX and DRV are still not enough to compete with WH. Upon surpassing the threshold for DRX, the dislocations annihilate (without recombining) toward the newly formed DRX grain boundaries [32]. Consequently, the rate of tensile stress diminishes, resulting in peak stress. Figure 4 exhibits the microstructure near the fracture surface under the tested conditions. Upon comparison with the initial microstructure depicted in Figure 2, it becomes evident that small fine DRX grains form a typical “necklace” feature, contributing significantly to dynamic softening in the flow curves. Beyond the peak stress, the tensile stress gradually decreases, which is attributed to the dynamic softening and hot damage caused by microvoids and microcracks. The dynamic softening intensifies with the increasing strain, particularly before the onset of local necking, which can effectively neutralize WH. On the other hand, microvoids initiate and expand with further growth in strain, resulting in the gradual occurrence and accumulation of internal hot damage. As a consequence, the effective bearing surface of the superalloy decreases, leading to necking [33,34]. However, a rapid decrease in flow stress indicates the onset of the local necking stage. Once local necking begins, the final rupture occurs quickly. In forming Inconel 625 superalloy parts, once the rupture occurs, it directly affects the performance of its parts. Thence, the plastic stage is the most important for the tensile curve of the tested superalloy.

In addition, from Figure 3, the deformed energy storage accumulates slowly at low tensile temperatures, impeding the evolution of DRV and DRX [35]. As a result, the flow stress of the superalloy rises with decreasing deformation temperature. For instance, at a strain rate of 0.005 s^−1^, the peak stress grows from 344.9 MPa to 522.4 MPa at 700 °C compared to 800 °C. However, at high strain rates, the deformation period shrinks, which restricts complete dislocation annihilation. Therefore, at the same deformation temperature, the flow stress diminishes with a decrease in strain rate. At 750 °C, the peak stress decreases from 529.2 MPa to 435.2 MPa at a strain rate of 0.01 s^−1^ compared to 0.001 s^−1^. However, when comparing the peak stress (386.5 MPa) of TA32 titanium alloy at 750 °C and 0.001 s^−1^ to that of Inconel 625 alloy [36], it becomes evident that Inconel 625 exhibits superior high-temperature tensile strength and a greater capacity to endure harsh environments.

Moreover, Figure 3 shows that the total elongation of the samples is abnormal under the fixed deformation temperature. This may be attributed to the significant imbalance among the WH, dynamic softening, and local necking stages. For instance, the flow curves in Figure 3a,b reveal that WH occurs for very short periods at 700 °C and 0.005 s^−1^, while long-term dynamic softening leads to premature fracture of the material at 700 °C and 0.01 s^−1^. Furthermore, thermal stress can destroy the binding force of the grain boundaries at high deformation temperatures. Accordingly, the weakened grain boundaries can be easily separated and function as the nucleation sites of microfractures or microvoids [33]. At lower strain rates (e.g., 0.005 s^−1^ and 0.001 s^−1^), sufficient time is afforded for the initiation of microfractures or microvoids. Consequently, lower plasticity and reduced elongation are observed at lower strain rates.

### 3.2. Fracture Characteristics

#### 3.2.1. Influences of Deformation Temperature

**Figure 5:**
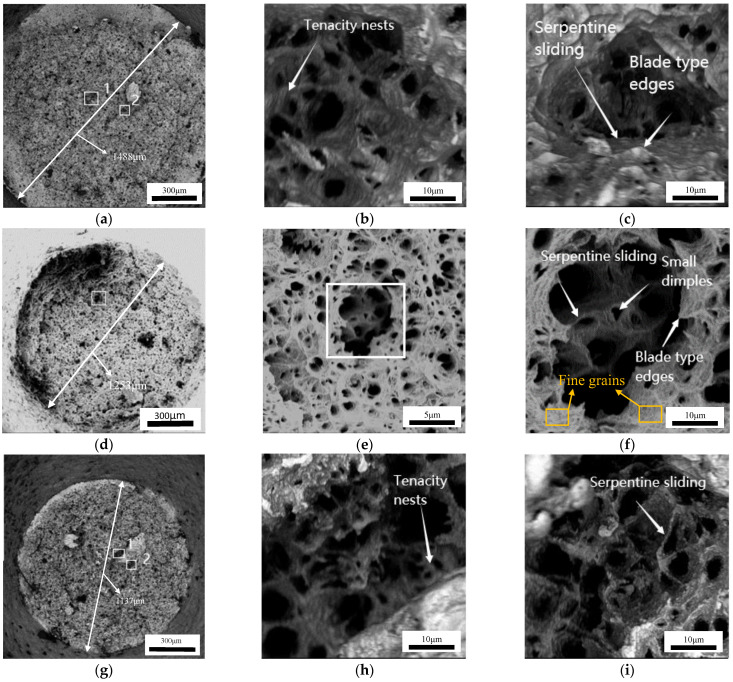
The fracture morphologies of the fracture samples under the strain rate of 0.001 s^−1^ and deformation temperature of: (**a**–**c**) 700 °C, (**d**–**f**) 750 °C, (**g**–**i**) 800 °C.

Figure 5 illustrates the fracture morphology of specimens subjected to temperatures of 700, 750, and 800 °C at a strain rate of 0.001 s^−1^, respectively. All fracture surfaces exhibit dimples, i.e., a ductile fracture mode. At 700, 750, and 800 °C, the measured fracture diameters are 1488 (Figure 5a), 1253 (Figure 5d), and 1137 μm (Figure 5g), respectively. Notably, the fracture diameter decreases with the rise in deformation temperature, i.e., the plasticity improves with the increase in hot deformation temperature. Moreover, the impact of the deformation temperature on fracture morphology is observed through specific magnified regions. Figure 5b,c display high-magnification dimple features corresponding to the items labeled 1 and 2 in Figure 5a. At 700 °C, small dimples are observed, accompanied by numerous blade-like edge features distributed across the larger dimples and tear edges. Thus, the numerous coalesced dimples form during thermal tensile deformation. Moreover, discernible tenacity nests can be seen along the walls of the large dimples (Figure 5b). Typically, the atomic diffusion within or near the grain boundaries generates tenacity nests facilitated by dislocation creep. These ductile nests signify inadequate plastic deformation, which hinders further necking contraction. Meanwhile, at low tensile temperatures, the necking diffusion capability is extremely limited, leading to the occurrence of mesoscopic damage. Thus, ductile fracture is the predominant fracture mechanism [37]. At 750 °C, the count of blade-like edges decreases, while the dimple enlarges compared to that at 700 °C. As depicted in Figure 5e,f, the large microvoids emerge at the fracture surface due to the coalescence of adjacent dimples, accompanied by residual small dimples at their base. The elevated deformation temperature stimulates dimple coalescence and microvoid formation [37]. The presence of finer grains indicates initiation of DRX (Figure 5f). In general, dislocations within the grains are alleviated through the growth of DRX grains, which induces a deviation in the atomic orientation arrangement and the formation of a porous grain structure, leading to intergranular fracture [38,39]. The elevated tensile temperature also promotes the thermal activation process, facilitating DRX behavior and resulting in an increased DRX fraction. The refined DRX grains and homogeneous microstructure that ensue act as inhibitors for microvoid coalescence and crack extension. So, the plastic deformation capability is improved at 750 °C. Figure 5h–i displays high-resolution images of 1 and 2 marked in Figure 5g. With increasing deformation temperature to 800 °C, a further enlargement in the size of the dimples is observed, accompanied by serpentine sliding features along the dimple walls. A specific degree of plastic deformation within the material leads to the formation of tough nests. As the deformation temperature rises, the prominence of the serpentine sliding feature intensifies. The elevated deformation temperature facilitates the activation of additional slip systems within the material, explicitly enhancing high-temperature plasticity [40].

#### 3.2.2. Influences of Strain Rate

**Figure 6:**
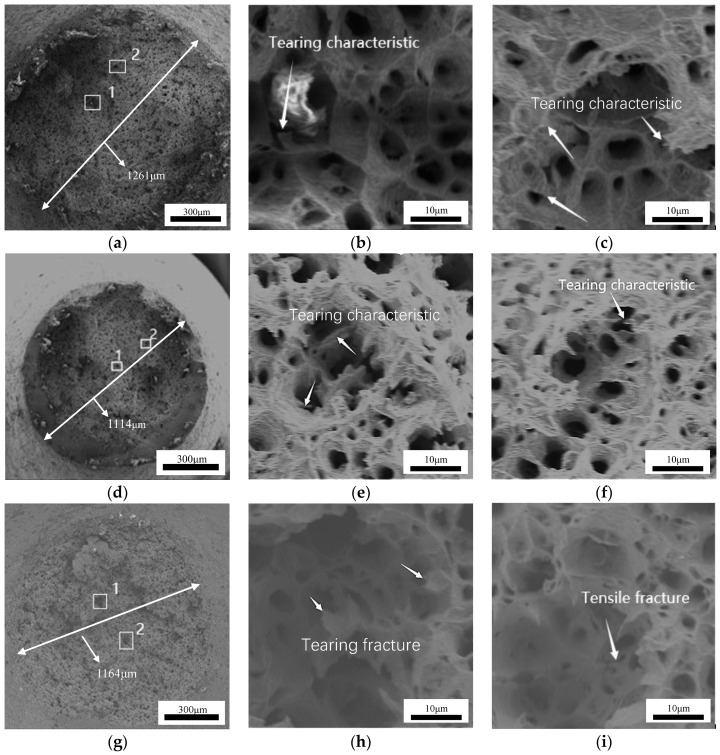
The fracture morphologies of the failure specimens under the deformation temperature of 750 °C and strain rates of: (**a**–**c**) 0.0005 s^−1^, (**d**–**f**) 0.005 s^−1^, (**g**–**i**) 0.01 s^−1^.

Figure 6 depicts the fracture images (750 °C) at strain rates of 0.0005, 0.005, and 0.01 s^−1^. At the three strain rates, the fracture morphology of the material is typical equiaxial dimple, and the corresponding section diameters are 1261 μm, 1141 μm, and 1164 μm, respectively. Generally, a small fracture section diameter indicates a good plastic deformation capacity [1]. The superior plastic deformation ability appears at 0.005 s^−1^ and 750 °C (consistent with the tensile rheological curve in Figure 3). Similar findings are noted in both the hot-rolled 2205/AH36 bimetal composite material [41] and the AA5083 aluminum alloy [42]. In addition, the dimple features are observed under the three strain rates, i.e., ductile fracture occurs. Figure 6b,c display high-magnification dimple features corresponding to the items labeled 1 and 2 in Figure 6a. As depicted in Figure 6b,c, a uniform distribution of large dimples can be observed across the fracture surface at a strain rate of 0.0005 s^−1^. Meanwhile, the knife-shaped edges induced by the connection of adjacent dimples are obvious. Figure 6e,f display high-magnification dimple features corresponding to the items labeled 1 and 2 in Figure 6d. As the strain rate increases to 0.005 s^−1^ (Figure 6d,e), a large number of knife-edge edges retain i.e., improved plastic deformation ability. However, significant fluctuations occur in the fracture surface at 0.01 s^−1^ due to extensive plastic deformation of the specimen during fracture, as evidenced in Figure 6h,i that display high-magnification dimple features corresponding to the items labeled 1 and 2 in Figure 6g. In the area with a low cross-section, the size of the dimple is small. At a high strain rate, the large flow stress during the tensile process causes the tearing of the dimple wall along the direction perpendicular to the tensile axis. In the area with a low cross-section, a large number of knife-shaped edges are distributed, and large cracks are formed. Considering local holes to bridge micro-cracks, the internal damage of the material is intensified, and the fracture of the alloy is easily induced. In addition, with an increasing strain rate, the degree of recrystallization decreases, and DRX behavior can aggravate the microvoid coalescence [43]. Therefore, the internal necking capability of the material and the progression of grain morphology collectively govern the initiation, growth, and bonding mechanisms of the microvoids, which finally induces the rupturing of the sample.

### 3.3. Constitutive Model of Hot Tensile Behavior

#### 3.3.1. Arrhenius Phenomenological Model

Generally, the influence of deformation parameters on the tensile behavior of metal materials at high temperatures can be quantitatively characterized by the Arrhenius equation [44], i.e.,

(1) $$\dot{\varepsilon }{=A}_{1}\sigma^{n_{1}^{\;}}\exp\left(\frac{Q}{RT}\right)\;\mathrm{for}\;\alpha \sigma <0.8$$

(2) $$\dot{\varepsilon }{=A}_{2}\exp\left(\beta \sigma \right)\exp\left(-\frac{Q}{RT}\right)\;\mathrm{for}\;\alpha \sigma >1.2$$

(3) $$\dot{\varepsilon }=A{\left[\sinh\left(\alpha \sigma \right)\right]}^{n}\exp\left(-\frac{Q}{RT}\right)\;\mathrm{for}\;\mathrm{all}$$

where $\dot{\varepsilon }$ represents the strain rate, σ denotes true stress (MPa), Q stands for the apparent activation energy, T signifies the deformation temperature, $A_{1}$ , $A_{2}$ , A , $n_{1}$ , n , α , and β are the material constants, are material constants, $\alpha =\beta /n_{1}$ and R represents the universal gas constant.

In addition, the Zener-Hollomon ( Z ) parameter is employed to represent the influence of strain rate and deformation temperature on the rheological behavior. Its expression is given as:

(4) $$Z=\dot{\varepsilon }\exp\left(\frac{Q}{RT}\right)$$

According to Equation (4), the flow stress ( σ ) can be expressed as:

(5) $$\sigma =\frac{1}{\alpha }\ln\left\{{\left(\frac{Z}{A}\right)}^{1/n}+{\left[{\left(\frac{Z}{A}\right)}^{2/n}+1\right]}^{1/2}\right\}$$

Taking the natural logarithm of Equations (1)–(4) yields,

(6) lnZ=lnA+nln[sinh(ασ)]

(7) $$\ln\sigma =\frac{\ln\dot{\varepsilon }}{n_{1}^{\;}}-\frac{\ln A_{1}}{n_{1}}+\frac{Q}{n_{1}RT}\;\alpha \sigma <0.8$$

(8) $$\sigma =\frac{\ln\dot{\varepsilon }}{\beta }-\frac{\ln A_{2}}{\beta }+\frac{Q}{\beta RT}\;\mathrm{for}\;\alpha \sigma >1.2$$

(9) $$\ln\left[\sinh\left(\alpha \sigma \right)\right]=\frac{Q}{nRT}-\frac{\ln A}{n}+\frac{\ln\dot{\varepsilon }}{n}\;\mathrm{for}\;\mathrm{all}$$

**Figure 7:**
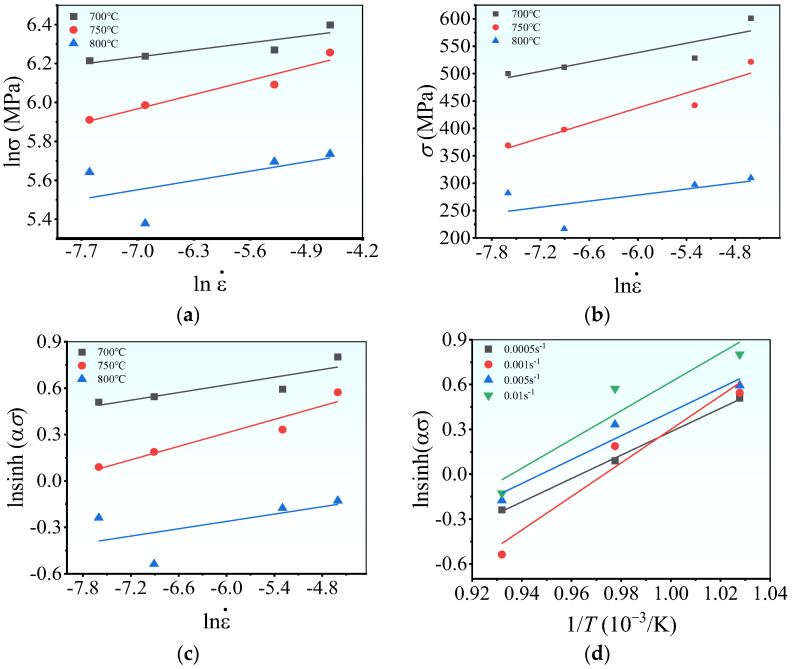
The relations of peak stress with the strain rate of: (**a**) $\ln\sigma -\ln\dot{\varepsilon }$ ; (**b**) $\sigma -\ln\dot{\varepsilon }$ ; (**c**) $\ln(\sinh(\alpha \sigma ))-\ln\dot{\varepsilon }$ ; (**d**) ln(sinh(ασ))−1/T .

Utilizing Equations (6)–(9), all material constants can be established via the regression analysis of the experimental data. Specifically, the peak stresses are initially utilized to estimate these material constants. The average values of $n_{1}$ and β can be derived from the $\ln\sigma -\ln\dot{\varepsilon }$ and $\sigma -\ln\dot{\varepsilon }$ plots (Figure 7a,b), which are 14.461 and 0.037, respectively. Thus, α can be calculated as 0.0026.

Based on the $\ln(\sinh(\alpha \sigma ))-\ln\dot{\varepsilon }$ (Figure 7c) and ln(sinh(ασ))−1/T (Figure 7d) plots, the values of n and Q can be 10.25 and 780.95 kJ/mol. Finally, the value of A can be determined as 4.5941 × 10^36^.

Subsequently, the constitutive model for the peak stress can be approximated as follows:

(10) $$\left\{ \begin{array}{l} \sigma_{p}=\frac{1}{0.0026}\ln\left\{{\left(\frac{Z}{\;4.594110^{36}}\right)}^{1/n}+{\left[{\left(\frac{Z}{\;4.594110^{36}}\right)}^{2/n}+1\right]}^{1/2}\right\}\\ Z=\dot{\varepsilon }\exp\left(\frac{780950}{RT}\right) \end{array}\right.$$

As previously reported, the parameters ( Q n , α and lnA ) exhibit nonlinear changes with increasing strain [45,46]. To accurately replicate the hot deformation behavior across the entire deformation range, the material constants are estimated by using the similar method in Figure 7. In this context, a strain interval of 0.05 is chosen, and fifth-order polynomial functions of strain are utilized to represent the correlation between the material constants and the true strain, as illustrated in Equation (11).

(11) $$\left\{ \begin{array}{l} \alpha =X_{11}\varepsilon^{5}+X_{12}\varepsilon^{4}+X_{13}\varepsilon^{3}+X_{14}\varepsilon^{2}+X_{15}\varepsilon +X_{16}\\ n=X_{21}\varepsilon^{5}+X_{22}\varepsilon^{4}+X_{23}\varepsilon^{3}+X_{24}\varepsilon^{2}+X_{25}\varepsilon +X_{26}\\ Q=X_{31}\varepsilon^{5}+X_{32}\varepsilon^{4}+X_{33}\varepsilon^{3}+X_{34}\varepsilon^{2}+X_{35}\varepsilon +X_{36}\\ \ln A=X_{41}\varepsilon^{5}+X_{42}\varepsilon^{4}+X_{43}\varepsilon^{3}+X_{44}\varepsilon^{2}+X_{45}\varepsilon +X_{46}\\ Z=\dot{\varepsilon }\exp\left(\frac{Q}{RT}\right)\\ \sigma =\frac{1}{\alpha }\ln\left\{{\left(\frac{Z}{A}\right)}^{1/n}+{\left[{\left(\frac{Z}{A}\right)}^{2/n}+1\right]}^{0.5}\right\} \end{array}\right.$$

**Figure 8:**
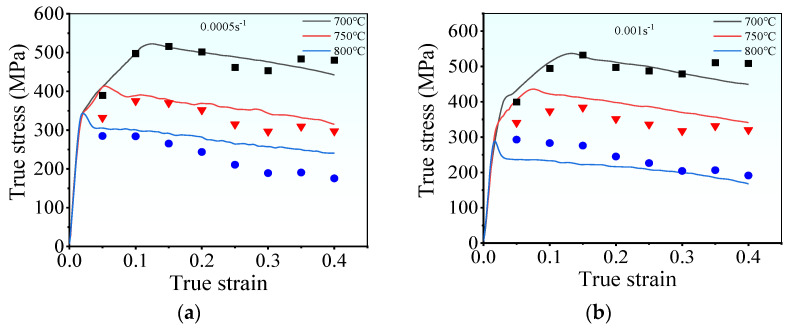

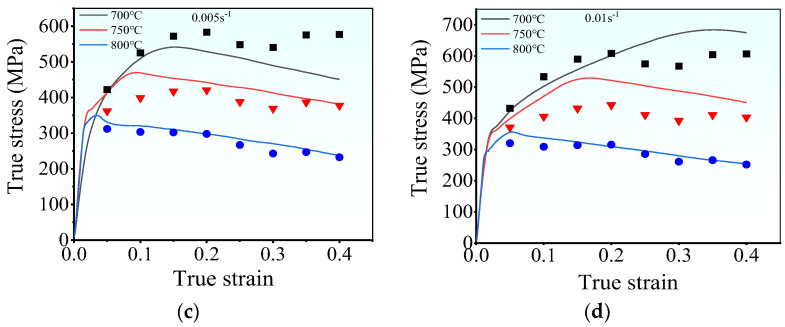
Comparisons of the predicted and experimental true stresses at various strain rates: (**a**) 0.0005 s^−1^, (**b**) 0.001 s^−1^, (**c**) 0.005 s^−1^, (**d**) 0.01 s^−1^.

Table 2 enumerates the values of $X_{ij}$ . Subsequently, the flow stress can be forecasted by using Equation (11). Figure 8 illustrates the comparison between the stress predicted by Equation (11) and the experimental stresses. For most of the hot deformation conditions, a good consistency is measured between the experimental true stress and the one predicted by the Arrhenius model. However, at a low strain rate (0.0005 s^−1^ and 0.001 s^−1^) or low deformation temperature (700 °C), a substantial discrepancy can be observed, which is attributed to the complex deformation mechanism of the superalloy. Usually, a low strain rate enables a long duration for the grain boundary migration and dislocation rearrangement, which contribute to sufficient dynamic softening. At low strain rates (Figure 8), there is notable competition between WH and DRV, resulting in an extended duration to reach the peak stress. Moreover, the dynamic softening is insufficient, and the rate of flow stress declines more than that under high deformation temperatures. Due to the complex hot deformation mechanism, the ability of the Arrhenius model (constructed based on empirical observations) is hindered. Thus, the prediction ability of the established Arrhenius model is reduced.

#### 3.3.2. PSO-BP Neural Network Model

As shown in Section 3.3.1, the accurately reconstructed high-temperature tensile behavior of the alloy poses challenges for the conventional Arrhenius phenomenological constitutive model. The ANN model can describe the thermal deformation characteristics of metals. Of these, the BP neural network possesses strong nonlinearity. It can predict the thermal deformation behavior of metals due to its flexibility, open hidden layer structure, high training efficiency, and strong self-learning and adaptability [47]. However, slow learning rates, susceptibility to local optima, and limited generalization capabilities are its limitations. In this case, the PSO algorithm can optimize the thresholds and weights within the ANN model. Eberhart and Kennedy first introduced PSO in 1995 [48]. This algorithm initializes a set of particles within the solution space (single particle—a potential optimal solution for problems involving extremum optimization). These particles possess three attributes: velocity, position, and fitness value. Governed by a set of rules, these particles exhibit adaptive behavior, adjusting their positions and velocities during each iteration to approach optimal spatial positions, seeking both individual and global extrema.

The fundamental PSO algorithm comprises velocity and position equations, which can be expressed by [49],

(12) $$v_{i}^{d}=wv_{i}^{d-1}+c_{1}r_{1}(pbest_{i}^{d}-x_{i}^{d})+c_{2}r_{2}(gbest^{d}-x_{i}^{d})$$

(13) $$x_{i}^{d+1}=x_{i}^{d}+v_{i}^{d}$$

Here, v denotes the particle renewal speed, d signifies the iteration times, $r_{1}$ and $r_{2}$ represent random numbers within the interval (0,1). w is the inertia weight, $c_{1}$ and $c_{2}$ denote the learning factors, $x_{i}$ signifies the particle, pbest represents the optimal individual of the previous generation, gbest denotes the swarm optimal individual.

**Figure 9:**
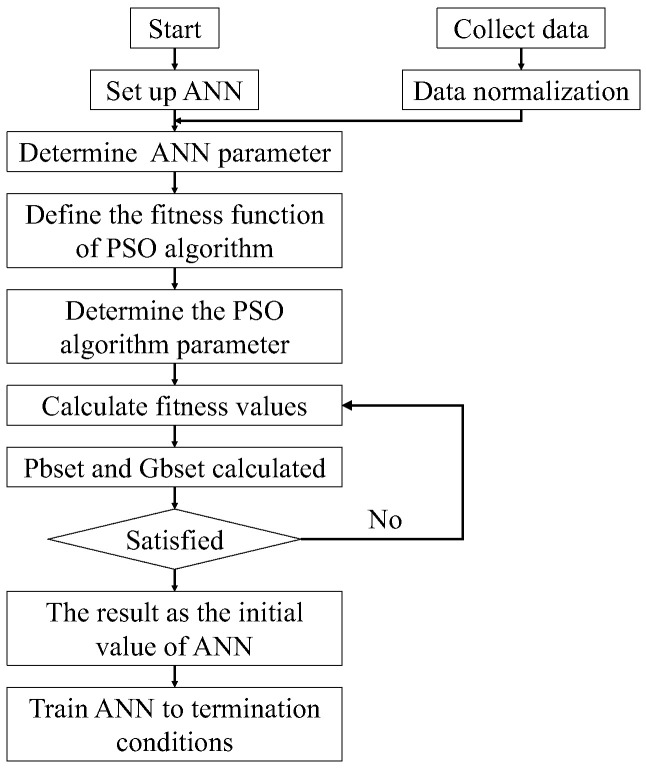
Framework of PSO-ANN model.

Here, a three-layer ANN model is first constructed. Temperature, strain rate, and strain are each denoted by three input neurons, while stress is represented by a singular output neuron. Following the principles of ANN theory, the neuron number is chosen to be 7 for the hidden layer [50]. In order to mitigate the influence of scale effects, the input and output data undergo normalization using the mapminmax function, constraining values within the range of [−1, 1]. The mapminmax function can be defined as follows:

(14) $$Y=\frac{2\left(X-X_{\min}\right)}{X_{\max}-X_{\min}}-1$$

where X is the input or the output variables, $X_{\max}$ and $X_{\min}$ represent the maximum and minimum values of X , respectively. Before constructing the ANN model, the true stress-strain curve of the studied superalloy is divided into 2400 segments with a strain interval of 0.002. Following this, 70% of the data is employed for training, 20% for validation, and the remaining portion for testing the capability of the established ANN model [51]. Subsequently, the PSO algorithm is utilized to optimize the ANN model, shown in Figure 9. The MATLAB R2021a toolbox is employed to train this model. Targeting an error of 10^−4^ and utilizing 1000 training epochs, the mutation probability, particle length, and population are set to 0.2, 50, and 100, respectively. Additionally, the maximum iteration times for the ANN are limited to 2000. The momentum factor is 0.8, the learning rate is 0.1, and the target error is set at 10^−3^.

**Figure 10:**
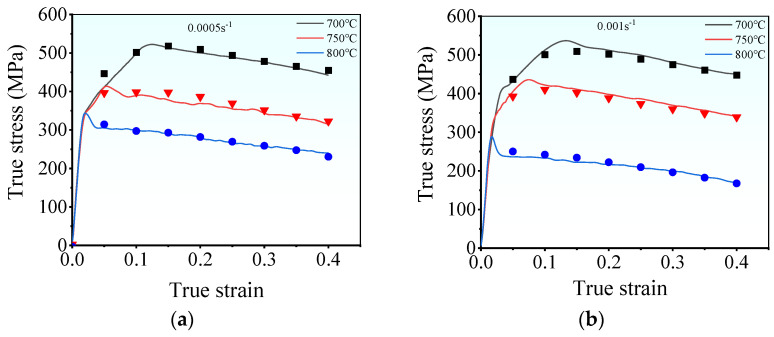

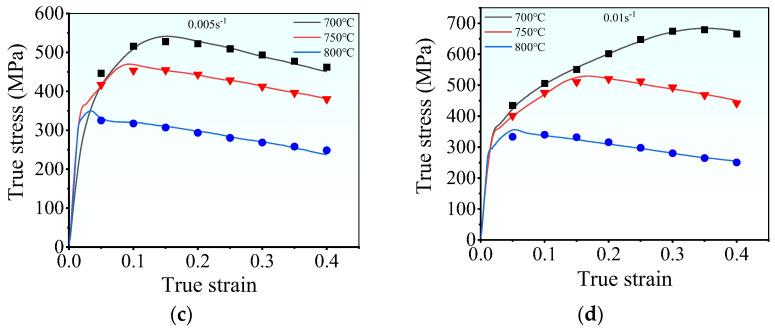
Comparisons of flow stress predicted by PSO-BP neural network and experimental ones at: (**a**) 0.0005 s^−1^, (**b**) 0.001 s^−1^, (**c**) 0.005 s^−1^, (**d**) 0.01 s^−1^.

Figure 10 compares the prediction of stress by the PSO-ANN model and experimental data, which reveals commendable alignments with the true strain and stress curves. Nevertheless, a minor deviation is observed at the onset of the prediction curve, which is due to the lack of specific physical mechanisms in the PSO-ANN model. Competitive behavior is observed among particles in the superalloy Inconel 625 during thermal deformation, which induces discontinuous yield in the initial deformation stage. This phenomenon poses a challenge in capturing accurate characteristics of the initial stage deformation.

#### 3.3.3. Accuracy Evaluation

To further assess the predictive ability of established models, two criteria, including the correlation coefficient ( R ) and relative absolute error ( AARE ), are employed, which are given as,

(15) $$R=\frac{\sum_{i=1}^{N}\left(X_{i}-\bar{X})(Y_{i}-\bar{Y}\right)}{\sqrt{\sum_{i=1}^{N}{\left(X_{i}-\bar{X}\right)}^{2}}\sqrt{\sum_{i=1}^{N}{\left(Y_{i}-\bar{Y}\right)}^{2}}}$$

(16) $$AARE=\frac{1}{N}\sum_{i=1}^{N}\left|\frac{Y_{i}-X_{i}}{X_{i}}\right|\times 100\%$$

where $X_{i}$ and $Y_{i}$ stand for the experimental and predicted values of flow stress, respectively. $\bar{X}$ and $\bar{Y}$ denote the averages of $X_{i}$ and $Y_{i}$ , respectively. N is the number of stress data.

**Figure 11:**
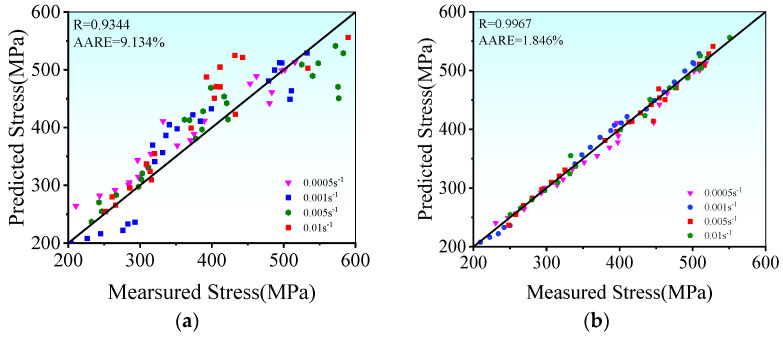
The correlations of the measured and forecasted true stresses by: (**a**) Arrhenius model; (**b**) PSO-ANN model.

Figure 11 illustrates a comparison of the correlation coefficients via two distinct methods. Clearly, the R values for the Arrhenius model and the PSO-ANN models stand at 0.9344 and 0.9967, respectively. This signifies that the hot tensile stress forecasted by the PSO-ANN aligns excellently with the experimental stress. Furthermore, the AARE across all tested conditions amounts to 9.13% for the established Arrhenius model and reduces to 1.85% for the PSO-ANN model. Hence, the predictive capability of the PSO-ANN model significantly surpasses that of the Arrhenius model.

## 4. Conclusions

In this study, the hot tensile deformation characteristics of the Inconel 625 alloy were investigated at a deformation temperature of 700–800 °C and a strain rate varying from 0.005 to 0.01 s^−1^. The impact of the deformation parameters was analyzed on the fracture morphology. Furthermore, a constitutive analysis was conducted using Arrhenius and PSO-ANN models, and the significant conclusions were outlined below:

(1) The high-temperature tensile flow stress of Inconel 625 was affected by temperature and strain rate. A reduction in temperature limited the accumulation of energy storage rate during tensile deformation, consequently decelerating DRV and DRX, which increased flow stress. Conversely, the reduced strain rate prolonged the tensile deformation duration, leading to the complete loss of dislocations and causing a decline in the flow stress.
(2) The alloy exhibited ductile fracture (primary mode) due to the combined effects of local necking and microvoid coalescence. However, the growth of DRX at a low strain rate and a high deformation temperature induced intergranular fractures.
(3) Two constitutive models, the Arrhenius and PSO-ANN models, were developed to forecast the hot tensile flow behaviors of the investigated superalloy. Findings reveal that the PSO-ANN model exhibited superior performance and predicted the hot deformation characteristics of the examined superalloy precisely.

## Figures and Tables

**Table 1:** Nominal compositions of the tested superalloy (wt. %).

Cr	Mo	Nb	Fe	Al	Ti	C	Mn	Si	Co	Ni
20	8	3.15	5	0.4	0.4	0.1	0.5	0.5	1	Bal.

**Table 2:** Calculated values of coefficient $X_{ij}$ in Equation (11).

$X_{ij}$	*i* = 1	*i* = 2	*i* = 3	*i* = 4
*j* = 1	−3.429	16,320	901,000	218,100
*j* = 2	3.81	−21,880	−1,465,000	−2,663,000
*j* = 3	−1.576	10,490	867,300	1,228,000
*j* = 4	0.3105	−2003	−228,800	−262,000
*j* = 5	−0.02804	70.08	25,710	24,690
*j* = 6	0.0034	22.21	−93.67	−637.7

## Data Availability

Data are contained within the article.
